# Disinfection by Hot Air

**Published:** 1883-03

**Authors:** 


### The Late Dr. Marshall H. Webb.

### A SUGGESTION.

From reading the eulogy on the late Dr. Marshall H. Webb in the Feburary No. of the *Dental Cosmos*, I am sorry to learn that he has left a widow and three children unprovided for, and that this is owing to the fact that he neglected his office practice, in order to teach others the art of filling teeth. As the late Dr.

